# Real life experience on the use of Remdesivir in patients admitted to COVID-19 in two referral Italian hospital: a propensity score matched analysis

**DOI:** 10.1038/s41598-024-59957-w

**Published:** 2024-04-23

**Authors:** Nicola Veronese, Francesco Di Gennaro, Luisa Frallonardo, Stefano Ciriminna, Roberta Papagni, Luca Carruba, Diletta Agnello, Giuseppina De Iaco, Nicolò De Gennaro, Giuseppina Di Franco, Liliana Naro, Gaetano Brindicci, Angelo Rizzo, Davide Fiore Bavaro, Maria Chiara Garlisi, Carmen Rita Santoro, Fabio Signorile, Flavia Balena, Pasquale Mansueto, Eugenio Milano, Lydia Giannitrapani, Deborah Fiordelisi, Michele Fabiano Mariani, Andrea Procopio, Rossana Lattanzio, Anna Licata, Laura Vernuccio, Simona Amodeo, Giacomo Guido, Francesco Vladimiro Segala, Mario Barbagallo, Annalisa Saracino

**Affiliations:** 1 Department of Precision and Regenerative Medicine and Ionian Area (DiMePRe-J), Clinic of Infectious Diseases, University of Bari “Aldo Moro”, Piazza Giulio Cesare N. 11 Cap, 70124 Bari, Italy; 2 Geriatrics Section, Department of Internal Medicine, University of Palermo, Palermo, Italy

**Keywords:** Infectious diseases, Medical research, Drug development

## Abstract

Remdesivir (RDV) was the first Food and Drug Administration (FDA)-approved medication for COVID-19, with discordant data on efficacy in reducing mortality risk and disease progression. In the context of a dynamic and rapidly changing pandemic landscape, the utilization of real-world evidence is of utmost importance. The objective of this study is to evaluate the impact of RDV on patients who have been admitted to two university referral hospitals in Italy due to COVID-19. All patients older than 18 years and hospitalized at two different universities (Bari and Palermo) were enrolled in this study. To minimize the effect of potential confounders, we used propensity score matching with one case (Remdesivir) and one control that never experienced this kind of intervention during hospitalization. Mortality was the primary outcome of our investigation, and it was recorded using death certificates and/or medical records. Severe COVID-19 was defined as admission to the intensive care unit or a qSOFAscore ≥ 2 or CURB65scores ≥ 3. After using propensity score matching, 365 patients taking Remdesivir and 365 controls were included. No significant differences emerged between the two groups in terms of mean age and percentage of females, while patients taking Remdesivir were less frequently active smokers (p < 0.0001). Moreover, the patients taking Remdesivir were less frequently vaccinated against COVID-19. All the other clinical, radiological, and pharmacological parameters were balanced between the two groups. The use of Remdesivir in our cohort was associated with a significantly lower risk of mortality during the follow-up period (HR 0.56; 95% CI 0.37–0.86; p = 0.007). Moreover, RDV was associated with a significantly lower incidence of non-invasive ventilation (OR 0.27; 95% CI 0.20–0.36). Furthermore, in the 365 patients taking Remdesivir, we observed two cases of mild renal failure requiring a reduction in the dosage of Remdesivir and two cases in which the physicians decided to interrupt Remdesivir for bradycardia and for QT elongation. Our study suggests that the use of Remdesivir in hospitalized COVID-19 patients is a safe therapy associated with improved clinical outcomes, including halving of mortality and with a reduction of around 75% of the risk of invasive ventilation. In a constantly changing COVID-19 scenario, ongoing research is necessary to tailor treatment decisions based on the latest scientific evidence and optimize patient outcomes.

## Introduction

According to the latest WHO data (August 7th, 2023), more than 780 million cases of SARS-CoV2 infection have been reported globally, and almost 7 million deaths related to the disease have been recorded since the appearance of the virus in the world scenario^1^.

Although a complete vaccination course is still considered highly effective in preventing hospitalization and severe forms^2^, several studies have reported reduced vaccine effectiveness in subjects, especially those infected with Omicron variants or immunocompromised ones^3–5^. Even now, pharmacological approaches remain crucial to reducing disease progression.

Remdesivir (RDV) was the first Food and Drug Administration (FDA) approved medication for COVID-19 in October 2020 for use in hospitalized adults and pediatric patients^6^ due to antiviral activity against SARS-CoV-2, inhibiting viral RNA-dependent RNA polymerases due to active nucleoside triphosphate (GS-443902)^7^. It has also previously been known as a possible therapy against filoviruses (Ebola viruses, Marburg virus), previous coronaviruses (SARS-CoV, MERS-CoV), paramyxoviruses, and pneumoviridae^8–10^.

Since the first phase of the pandemic administration of Remdesivir has seen many changes in terms of timing^11^, counter-indications^12^, and association with monoclonal antibodies or oral antivirals^13–16^.

There is a notable discrepancy in the available data about the utilization of Remdesivir. Multiple studies have demonstrated that Remdesivir generally decreases the duration of recovery without impacting mortality rates, the necessity for mechanical ventilation^17,18^, or the improvement of patients with moderate-to-severe COVID-19 who require oxygen therapy^19,20^.

However, this positive effect was not observed in patients requiring high-flow oxygen therapy. Conversely, alternative findings have indicated that Remdesivir can lead to a decrease in hospitalization duration, disease progression, and overall survival rates^21–23^.

As shown for other interventions^24^, the utilization of real-world evidence is of utmost importance in the context of a dynamic and rapidly changing pandemic landscape. This evidence plays a critical role in customizing therapeutic approaches, taking into account factors such as vaccination status and risk profiles. Additionally, it aids in clarifying the effectiveness of various treatments, thereby facilitating informed decision-making across diverse populations and clinical contexts.

The objective of this study is to evaluate the impact of RDV on patients who have been admitted to two university referral hospitals in Italy owing to COVID-19. This be achieved by employing a propensity score-matched methodology, which aims to minimize the influence of confounding variables and bias.

## Materials and methods

### Study population

All patients with more than 18 years and hospitalized in Internal Medicine or Geriatrics Wards from March 2020 to September 2022 in the University Hospital (Policlinico) ‘P. Giaccone’ in Palermo, Sicily, Italy^1^ and in the University Hospital Policlinico (Bari, Italy) were enrolled in this study. The study conducted in Palermo was approved by the Local Ethical Committee during the session of the 28th of April 2021 (number 04/2021) and in Bari 28 April 2020 (Study Code: 6357).

All methods were performed in accordance with the relevant guidelines and regulations (Declarations of Helsinki). Informed consent/consent to participate was obtained from each participant and/or their legal guardian(s) who received information verbally and read the information letter.

### Exposure: Remdesivir

Remdesivir was administered intravenously in: a. patients hospitalized for pneumonia due to COVID-19, needing non-invasive ventilation; b. patients having some comorbidities (e.g., type 2 diabetes, cancer) that can increase the risk of severe COVID-19 forms. These indications follow the national guidelines available at the time of inclusion^2^. According to local protocol, all the patients received 200 mg of Remdesivir on the first day, followed by 100 mg once daily for the subsequent 9 days, 4 days, or 2 days as a maintenance dose, for a total of 10, 5, or 3 days of treatment. All the patients received the first dose of Remdesivir within the first three days of disease. Since patients having severe renal (creatinine clearance < 30 ml/min) or hepatic failure (ALT > 5x) cannot take Remdesivir, they were excluded from the analyses. Finally, we also recorded potential side effects due to this medication.

### Outcomes: mortality and severe COVID-19

Mortality was the primary outcome of our investigation and it was recorded using death certificates and/or medical records^3^. Severe COVID-19 was defined as admission to intensive care unit, or a qSOFAscore ≥ 2 or CURB65scores ≥ 3. Briefly, the qSOFA (quick SOFA) is made by three different items, i.e., altered mental status, respiratory rate > 22, systolic blood pressure less than 100 mmHg, with a score over two indicating a higher risk due to sepsis^16^. The CURB-65 (Confusion, Urea, Respiratory rate, Blood pressure, and age over 65 years) is used for estimating mortality associated with pneumonia: a score of 3 or more indicates severe forms of pneumonia^17^.

### Confounders

For a better understanding of the association between the use of Remdesivir and the outcomes of interest, we included several factors, such as:

- Demographic characteristics, including age, gender, and smoking status.
- Comorbidities, including hypertension, dyslipidemia, type two diabetes mellitus, and obesity. These medical conditions were diagnosed using medical history, drug history, and laboratory measures recorded in the first four days of hospitalization.
- Signs and symptoms typical of COVID-19, such as fever, anosmia, etc. (including the presence of pneumonia) recorded at hospital admission.
- Other therapies, including the use of corticosteroids, heparins, and/or monoclonal antibodies.

### Statistical analysis

To minimize the effect of potential confounders, we used a propensity score matching with one case (Remdesivir) and one control that never experienced this kind of intervention during hospitalization. Since some factors (namely, smoking status, hypertension, obesity and diabetes) were not balanced between cases and controls, we added as covariates in our analyses.

Data on continuous variables were normally distributed according to the Kolmogorov–Smirnov test and therefore reported as means and standard deviation values (SD) for quantitative measures and percentages for the categorical variables, by use or not of Remdesivir. Levene’s test was used to test the homoscedasticity of variances and, if its assumption was violated, Welch’s ANOVA was used. P values were calculated using the Student’s T-test for continuous variables and the Mantel–Haenszel Chi-square test for categorical ones.

The association between the use of Remdesivir and mortality during the follow-up was made using a Cox’s regression analysis and reporting the findings as hazard ratios (HRs) with their 95% confidence intervals (95% CI). In these analyses, we included vaccination against COVID-19 as covariate since unbalanced despite the use of Remdesivir and significantly associated with the outcomes of interest. To test the robustness of our results, we made several sensitivity analyses analyzing the interaction Remdesivir by the factors included (e.g., dose and duration of Remdesivir treatment, gender, presence of any comorbidity, COVID-19 clinics and the use of other therapies). In the case of age, we used the median value (= 56 years) for dichotomizing this variable. Finally, the association between Remdesivir use and the use of non-invasive ventilation during hospitalization or severe COVID-19 was analyzed using a logistic binary regression analysis, with the data reported as odds ratios (ORs) with 95% CI.

All analyses were performed using the SPSS 26.0 for Windows (SPSS Inc., Chicago, Illinois). All statistical tests were two-tailed and statistical significance was assumed for a p-value < 0.05.

### Ethical approval statement and consent to participate

The study conducted in Palermo was approved by the Local Ethical Committee during the session of the 28th of April 2021 (number 04/2021) and in Bari 28 April 2020 (Study Code: 6357).

Informed consent was obtained from each participant and/or their legal guardian(s) that received information verbally and read the information letter.

## Results

The initial cohort included a total of 1883 patients hospitalized for COVID-19. Of them, 1070 used Remdesivir during the hospital stay. The 1070 participants taking Remdesivir differed in several clinical characteristics compared to the 813 controls, particularly regarding comorbidities and the presence of pneumonia radiologically identified (p < 0.0001 for all the comparisons). Therefore, a propensity score matching was proposed for better accounting of these baseline differences.

After using a propensity score matching, 365 patients taking Remdesivir and 365 controls were included. The majority of the patients used Remdesivir for ten days (n = 216), followed by 5 days (n = 126) and 3 days (n = 23). Table 1 shows the baseline characteristics. No significant differences emerged between the two groups in terms of mean age and percentage of females, whilst patients taking Remdesivir were less frequently active smokers (p < 0.0001). Regarding comorbidities, whilst no differences emerged for the presence of any comorbidity (p = 0.21), patients using Remdesivir were less significantly affected by hypertension and type 2 diabetes (p = 0.03 for both comparisons) or obesity (p = 0.005). Regarding COVID-19 symptomatology, we observed significant differences between Remdesivir and control groups in terms of anosmia and dysgeusia, both more frequent in the control group and fever more frequent in the Remdesivir group (Table 1). Moreover, the patients taking Remdesivir were less frequently vaccinated against COVID-19. All the other clinical, radiological, and pharmacological parameters were balanced between the two groups.

**Table 1:** Baseline characteristics by use or not of Remdesivir, after the matching using a propensity score.

Parameter	Controls (n = 365)	Remdesivir (n = 365)	p-value
Demographics			
Age (mean, SD)	55.4 (15.0)	56.2 (17.1)	0.52
Females (%)	59.7	52.6	0.06
Current smokers (%)	9.3	3.0	< 0.0001
Comorbidities			
Any comorbidity	47.1	52.1	0.21
Hypertension	48.8	40.3	0.03
Dyslipidemia	20.5	26.6	0.07
Type 2 diabetes	20.8	14.2	0.03
Obesity	16.4	9.0	0.005
COVID-19 clinics			
Dyspnea	36.7	28.5	0.02
Anosmia	4.9	6.6	0.43
Dysgeusia	18.1	28.5	0.001
Fever	58.9	76.2	< 0.0001
Cough	34.2	41.4	0.06
Gastrointestinal symptoms (%)	14.8	20.5	0.05
SpO2 < 92%	71.0	71.0	1.00
Presence of pneumonia	93.2	94.0	0.76
Vaccinated against COVID-19 (%)	62.5	24.9	< 0.0001
Other therapies			
Use of corticosteroids	72.6	81.6	0.05
Use of heparins	80.0	81.6	0.64
Use of monoclonal antibodies	8.9	11.8	0.25

**Figure 1:**
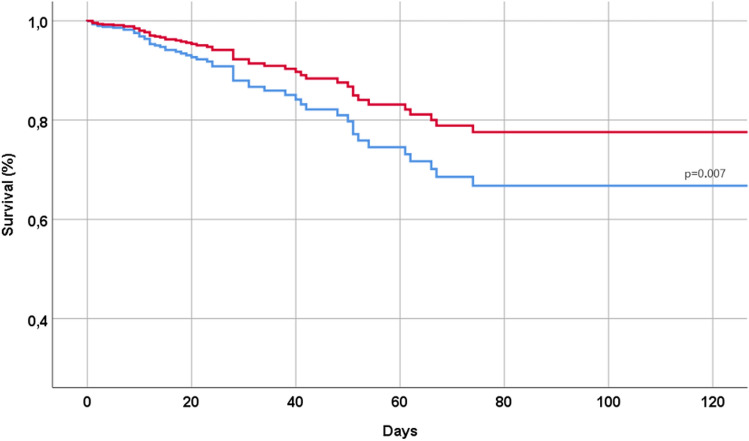
Association between use of Remdesivir and mortality during the follow-up period. In red patients taking Remdesivir, in blue controls. The analyses were made after matching using a propensity score our sample.

During a median follow-up time of 15 days (range 0 to 172), 58 patients (= 7.9% of the initial population) died, 490 (= 67.1%) used a non-invasive ventilation, and 571 (n = 78.2%) had a severe COVID. Table 2 shows the association between the use of Remdesivir and the outcomes of interest of our study. The use of Remdesivir, in our cohort, was associated with a significantly lower risk of mortality during the follow-up time (HR 0.56; 95% CI 0.37–0.86; p = 0.007), as also graphically reported in Fig. 1. Moreover, the use of Remdesivir was associated with a significantly lower incidence of a non-invasive ventilation (OR 0.27; 95% CI 0.20–0.36), but not with severe COVID (OR 1.09; 95% CI 0.78–1.52) (Table 2).

**Table 2:** Association between Remdesivir and outcomes of interest, after the matching using a propensity score.

Outcome	Cumulative incidence in controls	Cumulative incidence in Remdesivir	HR/OR, 95%CI	p-value
Mortality	11.2	4.7	0.56 (0.37–0.86)	0.007
Use of non-invasive ventilation during hospitalization	81.6	52.7	0.27 (0.20–0.36)	< 0.0001
Severe COVID^1^	85.5	71.0	1.09 (0.78–1.52)	0.61

^1^Severe COVID-19 was defined as qSOFA scores ≥ 2 or CURB-65 scores ≥ 3 or admission in intensive care unit. The results are reported as hazard ratios (HRs) with their 95% confidence intervals (CIs), after a propensity-score analysis, including vaccination status at the baseline and the smoking status, hypertension, obesity and diabetes since not balanced between Remdesivir and controls.

To test the robustness of our results, we did run several sensitivity analyses. Among all the factors analyzed, the use of Remdesivir decreased mortality in patients with dyslipidemia (HR 0.19; 95% CI 0.06–0.64) (p for interaction = 0.02) and in those having a SpO2 < 92% at the hospital admission (HR 0.30; 95% CI 0.11–0.81; p = 0.02) (p for interaction = 0.02). The interaction Remdesivir by other factors included did not reach the statistical significance (p for interaction > 0.05).

Finally, in the 365 patients taking Remdesivir, we observed two cases of mild renal failure requiring a reduction in the dosage of Remdesivir and two cases in which the physicians decided to interrupt Remdesivir for bradycardia and for QT elongation.

## Discussion

This study aimed to assess the efficacy of Remdesivir on clinical outcomes within a cohort of 712 patients admitted to the hospital due to COVID-19.

Our results demonstrate a robust correlation between the utilization of Remdesivir and positive outcomes in individuals hospitalized for COVID-19. Specifically, those who underwent Remdesivir treatment exhibited a nearly 50% reduction in the risk of mortality.

Our results diverge from other published evidence. Specifically, a recent meta-analysis indicates no association between the use of Remdesivir and a reduction in mortality risk^25,26^, supporting evidence from other authors that there is no impact of RDV on mortality risk^27^.

A major limitation of these studies, however, is that they did not consider timing of Remdesivir prescription from symptom onset^28^, which demonstrated to be a key driver of drug effectiveness and is today listed as a prescriptive criteria in the drug package insert^29^ and in all major international guidelines^30,31^.

In our study, all patients received the intervention drug within 3 days from symptom onset. Furthermore, another factor that could have influenced the difference in mortality is population heterogeneity in terms of clinical and demographic characteristics. In our study, utilizing propensity score analysis, two groups exhibit substantial overlap in numerous demographic aspects (age, gender) and clinical features (comorbidities, vaccination status, pneumonia, illness severity, oxygen saturation), as well as co-therapies (corticosteroids, heparin, monoclonal antibodies, and oral antivirals).

Moreover, in our population, the use of Remdesivir was associated with a reduction in disease progression with a lower incidence of non-invasive ventilation, with a reduction in these risks of almost 75%. This data is already discussed and well noted in other literature, confirming the hypothesis that Remdesivir mitigates the severity of the disease and reduces the need for aggressive respiratory support in hospitals^11,12,32^.

If we consider that the group taking Remdesivir was less frequently vaccinated for SARS-CoV-2, our conclusion about the efficacy of Remdesivir is much stronger. This is relevant in the light of evidence that places RDV as a drug with pan-variant activity, as shown in molecular surveillance studies^33,34^.

Regarding safety, we observed a low incidence of adverse events following the administration of RDV. We reported two cases in which modest renal failure necessitated dosage reduction, while in two other cases, bradycardia and QT prolongation led to discontinuation of Remdesivir. Although these adverse events are cause for concern, the overall safety profile of Remdesivir in our cohort appears to be acceptable. Furthermore, recent evidence among adverse effects of Remdesivir administration suggests that there is no association between acute kidney injury (AKI) and Remdesivir^16^; causes of AKI during Remdesivir administration could be attributed to the underlying SARS-CoV-2 infection^35,36^.

Overall, our study proved that Remdesivir is a well-tolerated treatment with rare adverse reactions, as demonstrated in other studies^37^.

We recognize some possible limitations to our study. As a retrospective observational study, it is susceptible to selection bias and unmeasured confounding variables. However, to overcome and address potential differences between the two groups (those receiving regimens with Remdesivir versus standard treatment), we conducted a propensity score analysis. This analytical approach effectively mitigates some biases due to the observational nature of the study^21^. The inclusion of a balanced comparison group aids in the generation of a more precise assessment of treatment effects by accounting for confounding variables^21^.

Second, the study was limited to a cohort of hospitalized COVID-19 patients, and the results may not be directly pertinent to other patient populations, including outpatients. Thirdly, the relatively small sample size and potential variation in treatment protocols across centers may limit the applicability of our findings. Finally, we could not use the Severe COVID Prediction Estimate (SCOPE)^38^ scores due to a lack of data on Interleukin 6 (IL-6) in several patients and the WHO COVID-19 severity classification to standardize outcomes.

In conclusion, our study suggests that the use of Remdesivir in hospitalized COVID-19 patients is associated with improved clinical outcomes, including halving of mortality and with a reduction of around 75% of the risk of invasive ventilation. These results are crucial, especially in a period when COVID-19 research is mostly focused on the management of high-risk patients and immunocompromised ones^39–41^ in greater need of effective therapies that reduce the risk of disease progression and mortality.

Additional real-life studies are required to confirm these findings and better comprehend all the potential benefits and hazards of Remdesivir in various patient subgroups, including the potential impact on COVID-19 sequelae^42,43^. In fact, with the constantly changing COVID-19 scenario, ongoing research is necessary to tailor treatment decisions based on the latest scientific evidence and optimize patient outcomes.

## Data Availability

The datasets used and/or analyzed during the current study are available from the corresponding author on reasonable request.
